# Cymatics for the cloaking of flexural vibrations in a structured plate

**DOI:** 10.1038/srep23929

**Published:** 2016-04-12

**Authors:** D. Misseroni, D. J. Colquitt, A. B. Movchan, N. V. Movchan, I. S. Jones

**Affiliations:** 1 DICAM, University of Trento, via Mesiano 77, I-38123 Trento, Italy; 2 Department of Mathematics, Imperial College London, South Kensington, London SW7 2AZ, UK; 3 Department of Mathematical Sciences, University of Liverpool, Liverpool L69 3BX, UK; 4 Mechanical Engineering and Materials Research Centre, Liverpool John Moores University, Liverpool L3 3AF, UK

## Abstract

Based on rigorous theoretical findings, we present a proof-of-concept design for a structured square cloak enclosing a void in an elastic lattice. We implement high-precision fabrication and experimental testing of an elastic invisibility cloak for flexural waves in a mechanical lattice. This is accompanied by verifications and numerical modelling performed through finite element simulations. The primary advantage of our square lattice cloak, over other designs, is the straightforward implementation and the ease of construction. The elastic lattice cloak, implemented experimentally, shows high efficiency.

## Introduction

In this paper we present a novel practical design and an experimental implementation of an approximate cloak in a structured flexural plate. It is based on the rigorous theoretical findings of Colquitt *et al*.^1^,^2^. In particular, for the lattice implementation, the cloaking transformation is not singular and hence does not require unrealistic infinite wave speed at the interior contour of the cloak. Since the first experimental demonstration of the microwave invisibility cloak^3^, there has been an explosion of theoretical and practical advances in the design and analysis of electromagnetic metamaterials^4^. In contrast, the significantly more challenging problem of creating invisibility cloaks and metamaterials for elastodynamics has been much less studied. Notable recent advances in the theoretical analysis of cloaks for elastic waves have been made by Milton *et al*.^5^,^6^, Norris & Shuvalov^7^, Brun *et al*.^8^,^9^, Farhat *et al*.^10^, Jones *et al*.^11^, Colquitt *et al*.^1^,^2^, Guenneau *et al*.^12^ and Parnell *et al*.^13^,^14^,^15^,^16^. These theoretical developments have been complemented with a series of experimental implementations of multi-scale mechanical cloaks performed by the group led by Wegener^17^,^18^,^19^.

In particular, the work by Milton *et al*.^5^,^6^ identifies the effects of ‘negative inertia’ in the elastic cloak and analyses the requisite strong anisotropy, not only in the elastic compliance, but also in the inertial properties of the cloak. The fact that, in the framework of Milton *et al*.^5^,^6^, the mass density of the material should behave as a tensorial quantity, rather than simply as a scalar, is an important and striking observation. A novel approach of dynamic homogenisation, capable of encapsulating such striking effects, was introduced and systematically studied by Craster and co-authors^20^,^21^. The asymptotic theory leads to an effective equation for the envelope function in the perturbation approximation relative to a standing wave associated with a periodic lattice or continuum structure. Although one cannot create an invisibility cloak for elastodynamics without recourse to a non-classical generalised theory of elasticity, Norris & Shuvalov^7^ later extended the framework of Milton *et al*.^5^,^6^ and showed that, with an appropriate choice of Gauge, one can choose between creating a micropolar elastic cloak or a cloak with tensorial density, for example. The concept, design, and theoretical analysis of elastodynamic invisibility cloaks are much more challenging compared to cloaks for membrane, acoustic, optical, and anti-plane shear waves, all of which are governed by the transformed Helmholtz equation. Although the theoretical framework for elastic cloaks is well defined, to the best of our knowledge its experimental implementation has never been successfully achieved for dynamic vector problems of elasticity. The pioneering work by Wegener and his group for Kirchhoff-Love plates^17^ is the only significant experimental contribution in this extremely challenging area. In a different context, multi-scale resonators were discussed^22^ in relation to an approximate cloaking referred to as “Directional cloaking” for elastic plates containing voids.

The elastic Kirchhoff-Love plate provides an efficient and rigorous framework for the analysis of elastic waves in thin plates. The propagation of flexural waves in a Kirchhoff-Love plate is governed by the biharmonic operator and waves in an homogeneous isotropic plate can be expressed as a linear combination of solutions of the Helmholtz equation (which we refer to as “membrane waves”) and solutions of the modified Helmholtz equation. The latter are evanescent fields but, nevertheless, may make a significant contribution through the boundary conditions and hence play a crucial role in the dispersion of flexural waves in structured plates^23^,^24^,^25^.

The design of the cloak presented here is distinct from the cloaks used by Wegener *et al*.^17^ and Chen *et al*.^26^, in so far as the cloak designed and constructed in the present paper is matched with a multi-scale mechanical structure, as opposed to a homogeneous continuous ambient matrix. Moreover, the cloak which we use is not singular, i.e. it does not require infinite wave speeds on the interior boundary; it is based on the regularised cloaking transformation and theoretical design by Colquitt *et al*.^1^ for membrane waves and later for flexural waves in plates^2^. We emphasise that the analysis and theoretical design presented in^2^ is two-dimensional; in contrast, the cloak design and numerical implementation described here is fully three-dimensional and corresponds precisely with the experimental parameters used, including the presence of non-negligible dissipative effects.

**Figure 1:**
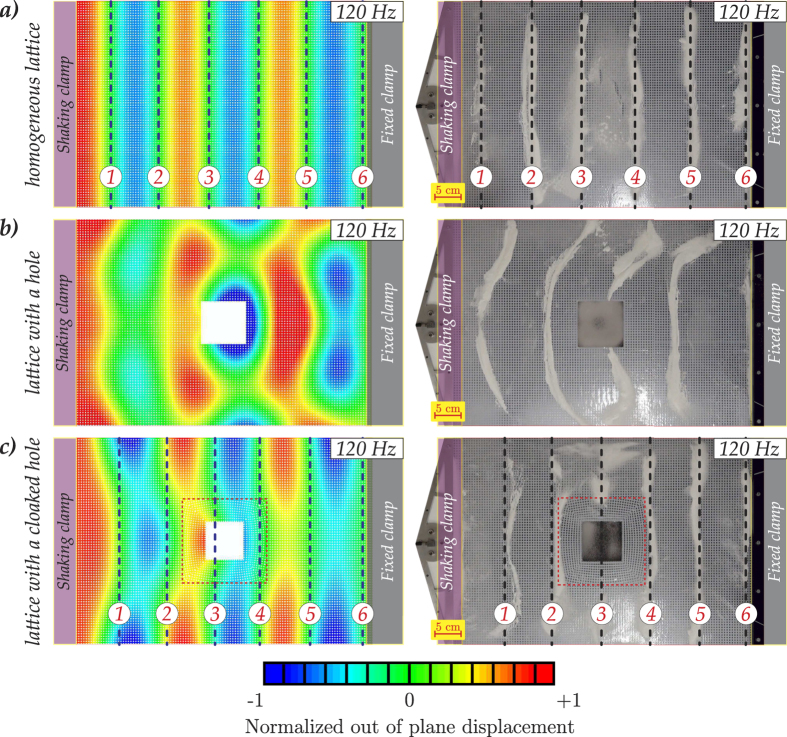
Comparison between experiments and numerical simulations in the case of an applied displacement with a frequency of 120 Hz. In the subfigures the dashed/black lines show the positions of the nodal lines of the vibrating plate. In the vicinity of the shaking clamp there cannot be any nodal lines because the edge is vibrating. Any powder adjacent to the shaking clamp does not represent a physical nodal line, and it is due to the clamp restricting the movement of the powder.

In Fig. 1 we show an example of the numerical and experimental implementations of the cloak design. These demonstrate the efficiency of the invisibility cloak comparing a uniform vibrating plate without a hole, a plate with an uncloaked hole, and a plate with a hole surrounded by the cloak – the latter reduces significantly the scattered field, as predicted.

It is emphasised that here we consider the full elastodynamical problem of wave propagation in a discrete metamaterial lattice cloak embedded within a multi-scale ambient medium; this should be distinguished from the earlier work of Wegener *et al*. on mechanical lattice cloaks^18^,^19^ wherein the static cloaking problem is considered and no waves propagate within the system.

The structure of the paper is as follows. We first advocate the idea of the Hooke-Chladni-Faraday visualisation, which is extremely efficient for the case of elastic structures such as flexural plates. We then present the proof of concept for the square cloak. This includes computational and experimental implementations, followed by a discussion of the experimental design and main results. We also discuss the concept of the regularised cloaking transformations, and the lattice approximation. Finally, we draw together important concluding remarks.

### The Hooke-Chladni-Faraday visualisation

Before presenting the experimental implementation and reviewing the mathematical model of the cloak, we first discuss an elegant technique that we will employ to visualise the time-harmonic vibration of plates. The method, known as the *“Hooke-Chladni-Faraday”* technique, has been utilised by many researchers in physics and mechanics for almost four centuries and continues to be used today. Indeed, the recent paper^27^ examines the visualisation of standing waves in dynamically reconfigurable liquid-based templates, which were used to assemble micro-scale materials into ordered structures with desired properties. This novel approach at a micro-scale level employed the idea of Faraday waves. A three-dimensional visualisation of acoustic standing waves was reported in a recent paper^28^ that demonstrated the elegance of the classical Hooke-Chladni-Faraday method, which continues to generate new ideas and exciting results. In particular, the experiment reported in^28^, shows levitating micro-particles along the nodal lines of a three-dimensional standing wave.

Much earlier than the time of Michael Faraday, Robert Hooke and then Ernst Chladni discovered an ingenious method to visualise standing waves in elastodynamics. This was especially effective for flexural resonances in elastic plates and membranes, as described in Chladni’s book^29^. The technique consists of drawing a violin bow over a metallic or glass plate that is covered with flour. Once the plate reaches a resonance, the flour collects along the nodal lines of the resonant mode providing an elegant yet efficient visualisation of the standing wave present in the plate.

Following Chladni’s experimental demonstration, the eigenvalue problem for the free vibrations of a square plate with free edges has been studied by many scientists, most notably, Lord Rayleigh^30^,^31^ and Ritz^32^,^33^ during the development of the now well known Rayleigh-Ritz method. Although no closed form solution currently exists, Ritz^33^, over a century ago, was able to construct remarkably accurate approximate solutions for the eigenfrequencies and nodal patterns. These classical studies have led to the fascinating area of Cymatics, which analyses methods of making sound and vibration visible and has attracted the attention of engineers, mathematicians, physicists and musicians around the world.

**Figure 2:**
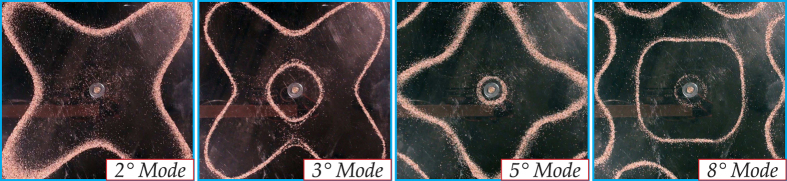
Hooke-Chladni-Faraday visualization of four eigenmodes of a square elastic plate with a free boundary.

For the purpose of illustration, we include in Fig. 2 examples of the Chladni patterns for eigenmodes of a square elastic plate with a free boundary, which are accurate and fully consistent with analytical findings. The patterns depend on the boundary conditions and on any inhomogeneities that may occur in the plate, such as voids or inclusions. In particular, Chladni patterns were never constructed for a plate with a hole surrounded by a structured cloak.

In the present paper, we visualise Hooke-Chladni-Faraday patterns for flexural waves around an obstacle surrounded by a multi-scale structured cloak and elegantly illustrate the efficacy of the cloak. An ABAQUS simulation for the mechanical configuration, identical to the one used in the experiment, provides accurate numerical data on the displacement amplitudes and stress distribution. We present the experimental visualisation to confirm the predicted wavefront profiles.

**Figure 3:**
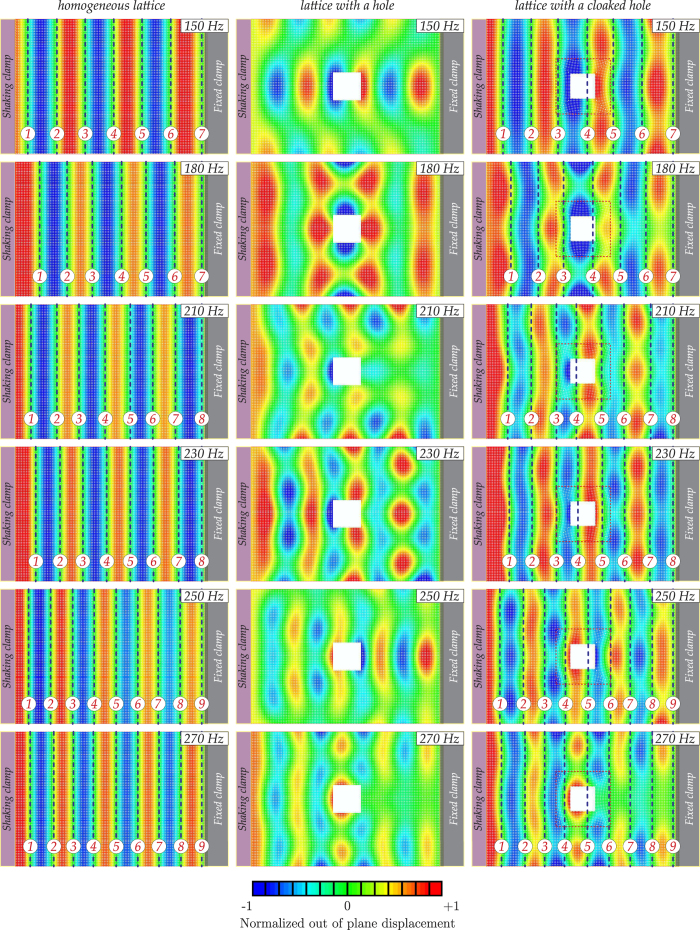
ABAQUS simulations performed in the case of a plate without a hole (left), a plate with an uncloaked hole (middle), and a plate with a hole surrounded by the cloak (right). For a frequency up to 250 Hz, the simulations show that the cloak reduces significantly the scattered field. The case of the frequency of 120 Hz is reported in the next figure together with the experiments. For frequencies higher than 250 Hz, (for example the lowest subfigure, showing 270 Hz), we can observe that the cloak starts to work less efficiently. In fact, while the reflected flexural wave field is preserved, there is scattering in the transmitted flexural wave beyond the cloak; this scattering is essentially absent for lower frequencies. In the subfigures the dashed/black lines show the positions of the nodal lines of the vibrating plate.

Compared to the classical settings for the frequency response problem for a rectangular Kirchhoff-Love plate mentioned above, we go further and consider a structured plate with a cloaked hole. Figure 1 includes Hooke-Chladni-Faraday patterns used for visualisation of the cloaking effect for flexural waves. These observations are new and demonstrate scattering patterns for three configurations, which include (a) a rectangular lattice-type plate, (b) a plate with a square hole, and (c) a plate with a structured cloak enclosing the hole. As in the original experiments by Hooke, the powder collects along the nodal lines of the vibrating plate thus indicating the position of the wavefronts, i.e. the locus of points on the wave with the same phase and zero displacement. The Hooke-Chladni-Faraday patterns allow us to conveniently visualise the wave front in the structured plate and, in particular, the cloaking effect for the case shown in Fig. 1(c). A detailed discussion of the experiment is given in the text below. Results of numerical simulations for several cases, corresponding to different frequencies of the incident wave, are reported in Fig. 3.

### Square cloak in an elastic plate: proof of concept

Here we present, for the first time, a proof of concept based on the cloaking of flexural waves in an elastic flexural plate by a structured square cloak.

We have performed both three-dimensional finite element (FE) computations, in ABAQUS, and also experiments to verify the efficacy of the structured square cloak in reducing the scattering of flexural waves by a void. The structured cloak has been designed and implemented in SOLIDWORKS. Each elastic ligament has a specified variable cross section that provides the required rigidities *D*_1_ and *D*_2_ (see equation (5)), first derived in the paper^2^ which addressed the corresponding two-dimensional model. The physical three-dimensional lattice cloak was created by milling holes into a polycarbonate plate; both ABAQUS and the milling machine were programmed using the same SOLIDWORKS original code. In so doing, experiments and simulations have identical three-dimensional geometry, material parameters, constraints and applied out-of-plane vibrations. Figure 5 illustrates both the lattice geometries implemented in ABAQUS and in the experiment. In the cloaked configuration (see Figs 1 and 3), we observe a significant reduction in the scattered field and, in particular, the reduction in the shadow region behind the scatterer and the restoration of the incident field represented by a plane wave.

### Numerical simulation

We have compared the wave field for three cases: the first for a homogeneous lattice in the absence of any void, the second in the presence of a void, and the third in the presence of a void surrounded by our specially designed invisibility cloak. The simulations have been performed using a parametric python script for ABAQUS, run by means of MATLAB.

We have computed the steady-state frequency response of each lattice using the Dynamic/Explicit package implemented in ABAQUS. Since the elastic lattices are constructed from thin elastic ligaments, we have performed the simulations employing 3D beam elements.

The regularised non-singular cloak design, presented here, provides a good approximation to an ideal theoretical cloak, as demonstrated in the experiment and in the corresponding three-dimensional numerical simulations. An out-of-plane flexural vibration of up to 250 Hz has shown the cloaking action. In particular, in Fig. 3 we show several cases of different frequencies, here the presence of the cloak leads to the plate with a void behaving in a similar way to an homogenous lattice without a void, i.e. the shadow region behind the void has been significantly reduced.

### The experimental implementation

**Figure 4:**
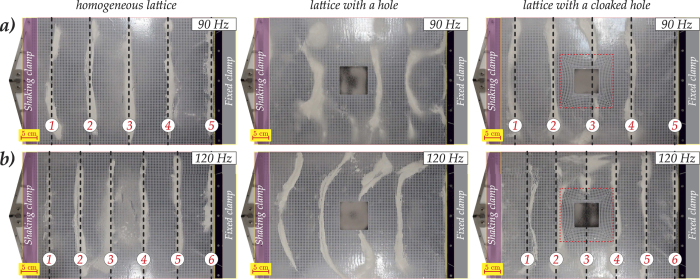
The cymatics experiments. Two cases are presented for different frequencies: 90 Hz (**a**) and 120 Hz (**b**). We show the profile of the nodal lines (wavefronts) for the case of a flexural plate without a hole, a plate with an uncloaked hole, and a plate with the hole surrounded by the lattice cloak. For both frequencies, the reduction of the perturbation of the wavefronts, due to the cloaking action, is observed.

We use the Hooke-Chladni-Faraday visualisation described above in order to demonstrate and analyse qualitatively the cloaking effect. In Fig. 4 we show two cases of cymatic visualisation of the lattice cloaking for two different frequencies of 90 Hz and 120 Hz. These are presented for three different flexural configurations, described below, and illustrated by a movie in the supplementary material. The cloaking action is shown in the form of restored wavefronts behind the hole surrounded by the lattice cloak.

Three structured flexural systems, namely a homogenous lattice, a lattice with a hole, and a lattice with a cloaked hole, have been produced by drilling polycarbonate plates (white 2099 Makrolon UV from Bayer) with an EGX-600 Engraving Machine (accuracy 0.01 mm, manufactured by Roland). The mechanical properties of the polycarbonate, namely elastic modulus, mass density and Poisson’s ratio, are respectively *E* = 2350 MPa, *ρ* = 1200 kg/m^3^ and *v* = 0.35. The total damping observed in the experiment is comprised of an internal damping (intrinsic viscosity of the polycarbonate) and of an external damping (air). We have measured the value of the decay exponent by performing an auxiliary experiment on a polycarbonate plate given an initial shake at a resonant frequency for a finite time interval and measuring the decay time by an accelerometer mounted on the plate. We have quantified the decay exponent to be equal to 0.0234 *s*^−1^. This dissipation has been accounted for in the ABAQUS simulation accordingly.

**Figure 5:**
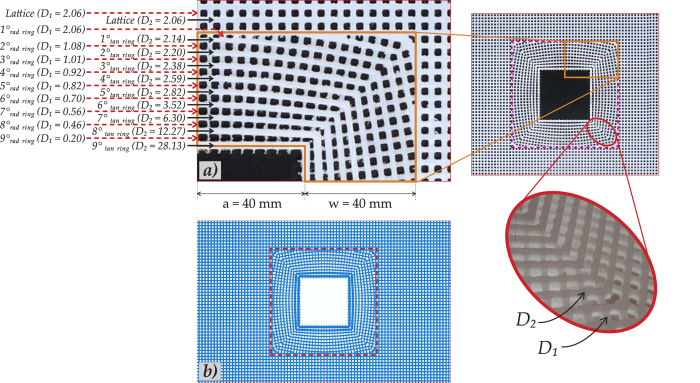
(**a**) The geometry of the real cloak which was milled from a sheet of polycarbonate; (**b**) a frontal view of the geometry implemented in ABAQUS. The magnified region of part (**a**) shows the physical realisation of the square cloak embedded in a lattice. By observing the enlarged detail of the square cloak, the different cross-sectional areas of the ligaments are clearly visible and chosen to match the required *D*_1_ and *D*_2_ principal flexural rigidities (see (5)). In the subfigure (**a**), the specific values for the rigidities are reported. The geometrical parameters from (3) are chosen as follows *w* = 40 mm, *a* = 40 mm and *ε* = 0.25.

The lattice cloak as well as the geometrical and material parameters were chosen consistently, according to the same data set stored in the SOLIDWORKS file, as illustrated by the table in Fig. 5(a). Thin transparent film was used to cover the flexural lattice system to enable the use of powder for the Hooke-Chladni-Faraday visualisation.

The lattices, externally measuring 600 mm by 400 mm, have ligaments of constant cross-section (1.75 mm by 2.45 mm) outside the cloak, whereas within the cloak the ligaments have variable width and height according to the analytical algorithm as reported in the final section of this paper (pp. 8–10). All the parameters of the structured cloak are reported in Fig. 5.

**Figure 6:**
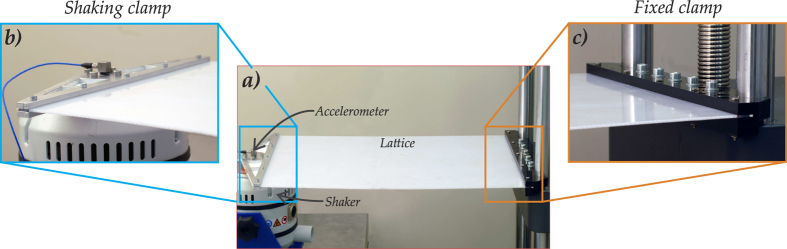
The vibration apparatus employed in the experiments (a) and the details of the constraints on both sides of the plate, namely a shaking clamp (b) and a rigid clamp (c).

The lattices, constrained by clamps on the two shorter sides and having the other sides free, have been excited by using a TIRA Vibrations Test System TV51144 and BOSE ElectroForce 3300 Series II, connected to the left clamp; the experimental arrangement is shown in Fig. 6. The maximum amplitude (1 mm peak to peak) of the sinusoidal displacement has been imposed by the oscillating clamp connecting the Vibrations Test System to an NI CompactRIO system, interfaced with LabVIEW 2014 (National Instruments).

### Effectiveness of the cloak and comparison with the computational model

The qualitative assessment of the effect of the cloak has been carried out using the Hooke-Chladni-Faraday technique which shows the positions of the nodal lines of the vibrating plate.

The boundary conditions were chosen consistently, both in the numerical simulation, and the physical experiment: one side of the plate is rigidly clamped, while the opposite side is attached to a moving clamp and excited by applying a time-harmonic displacement. The remaining two sides of the plate, interior boundary of the cloak, and the boundary of the square hole are traction free, i.e. the bending moments and the transverse forces are equal to zero at these boundaries.

Figure 1 shows the comparison between the experiment and the numerical simulations in the case of an applied displacement with a frequency of 120 Hz. The nodal lines are highlighted by dashed lines in subfigures (a,c). It is clear that, in the case of the homogeneous lattice and the lattice with a cloaked hole, the nodal lines can be identified while, in the case of a lattice with an uncloaked hole, identifying the nodal lines is significantly more difficult for both the ABAQUS simulations and the real experiments. It is important to remark that, since the clamp on the left-hand side is oscillating, the left-hand boundary of the plate cannot be a nodal line. Hence, the presence of any powder in this region does not represent a physical nodal line, and it is due to the clamp restricting the movement of the powder.

In the upper part of Fig. 1 we see the unperturbed plate without a hole, with a one-dimensional frequency response pattern clearly evident. In the middle part of Fig. 1 we show the plate containing an uncloaked square void, with a free boundary. We observe similar shadow regions and consistency in terms of the number of nodal lines both, in the ABAQUS simulation, and the physical experiment with a real structured plate. Finally, we demonstrate in the lower part of Fig. 1 that the shadow region is significantly reduced when the invisibility cloak, depicted in Fig. 5a, is introduced around the void so that the wave pattern outside the hole becomes almost planar, as expected. It is clear that there is good qualitative agreement between the experimental and numerical results. In fact, in the case of a cloaked void, the nodal lines showing the incident field represented by a plane wave are almost straight, similar to the case of the homogeneous lattice. On the other hand, in the absence of the cloak, the nodal lines appear to be strongly influenced by the hole. In this case we observe rounded nodal lines that differ significantly from the straight wavefronts observed for the homogenous lattice. From Fig. 3, we conclude that for frequencies higher than 250 Hz, (for example the lower subfigure of 270 Hz), the cloak starts to work less efficiently. In fact, while the reflected flexural wave field is preserved, there is scattering in the transmitted flexural wave beyond the cloak, almost totally absent for lower frequencies.

In the present paper we demonstrate the cloaking effect for wavefronts parallel to the sides of the cloak however, it has been demonstrated that the cloaking effect persists over all angles of incidence, for both membrane^1^ and flexural^2^ waves in addition to cylindrical waves.

**Figure 7:**
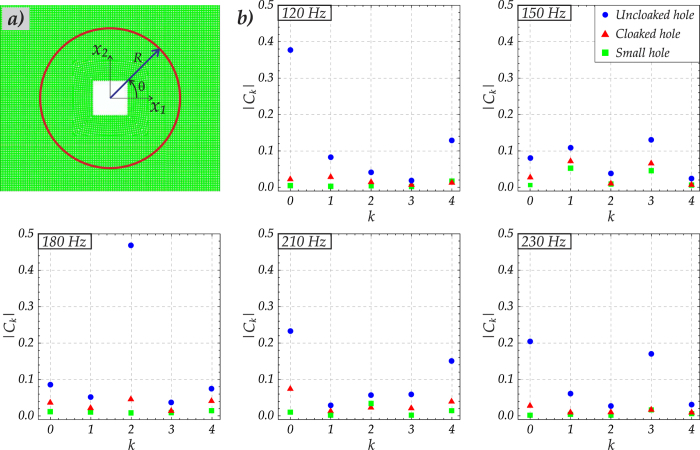
Fourier coefficients for the scattered fields around the hole and the cloaked hole in the flexural lattice. (**a**) A scattered field is measured on a circle of fixed radius *R* = 150 mm, centred on the origin; the circle surrounds the square hole; the same circle is used for the uncloaked hole of the size 80 mm, the cloaked hole of the same size, and the small hole of the size 20 mm; the regularisation parameter is chosen as *ε* = 0.25. (**b**) The five diagrams show the moduli of the Fourier coefficients (measured in [mm]) in the representations of the scattered fields at the frequencies of 120 Hz, 150 Hz, 180 Hz, 210 Hz and 230 Hz; the cloaking action is clearly visible by comparison of relatively large coefficients for the case of an uncloaked hole with the significantly reduced coefficients for the case of the cloaked hole.

The quantitative data, characterising the effectiveness of the cloak, is presented as the set of Fourier coefficients *C*_*k*_, measured for the scattered fields on a circle of a sufficiently large radius (as shown in Fig. 7), around the uncloaked and cloaked holes (the size of both holes is 80 mm) and compared with the reference Fourier coefficients, corresponding to a plate with a small hole (of the size 20 mm); these correspond to the non-dimensional small parameter *ε* = 0.25 (chosen according to the experimental setup) employed in the formula (3) below. For each of three cases, (a) uncloaked hole, (b) cloaked hole and (c) the plate with a small hole, the scattered fields are evaluated on the same fixed circle (see Fig. 7(a)), of radius *R* = 150 mm, as functions of the polar angle *θ*. A Fourier expansion in the standard complex form is used for the scattered field on the specified circle:


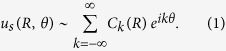


Due to the symmetry of the geometry of the plate and of the applied load, with respect to the *x*_1_-axis, the coefficients *C*_*k*_ in (1) are real, and *C*_*k*_ = *C*_*−k*_. Figure 7(b) gives a graphical illustration of the comparison between the three sets of Fourier coefficients for five cases of frequencies of 120 Hz, 150 Hz, 180 Hz, 210 Hz and 230 Hz. We show that the moduli of the Fourier coefficients in the representation of the scattered field around an uncloaked finite hole are larger than the moduli of the Fourier coefficients for the scattered fields around a cloaked hole of size *a* and around a hole of a small size *εa*. We also note that the coefficients *C*_*k*_ have the physical dimension of Length, and the measurements reported in Fig. 7(b) are in [mm].

A significant reduction in the magnitude of the Fourier coefficients in the case of a cloaked hole is observed compared to the case of the uncloaked hole. To be more precise, the coefficients generated for the scattered field around the cloaked hole are sufficiently close to those obtained for the scattered field around the small hole.

### Theoretical framework of the square cloak

In most of the papers addressing the design of cloaks for linear waves, a singular radially symmetric push-out transformation is employed (see, for example^13^,^34^) which maps a point to a finite disc. For theoretical and computational models of continuous media, such an approach is adequate. However, for practical implementations it poses substantial difficulties. In particular, not only does the transformation lead to infinite wave speeds on the interior boundary of the cloak, but also the required cloaking material is characterised by an unrealistic strong anisotropy. Indeed a lattice structure, instead of a continuum, can be used to create an invisibility cloak. A significant advantage of lattices is that they naturally accommodate high contrasts in their compliance leading to strong anisotropy. However, a radial discrete cloak that fits inside a circular ring would not match any periodic lattice, and the presence of a geometrical mis-match on the interface boundary leads to a substantial mis-match in the interface boundary conditions.

For the design presented here, we choose to employ a square cloak following the theoretical framework^2^. The cloak is embedded in an ambient square lattice, which is subjected to out-of-plane flexural vibrations. We consider a thin structured plate with a defect represented by a void with a free boundary, which significantly influences the wave field, as illustrated in Fig. 3 structured cloak^1^,^2^ is then installed around the void.

### The regularised cloaking transformation

**Figure 8:**
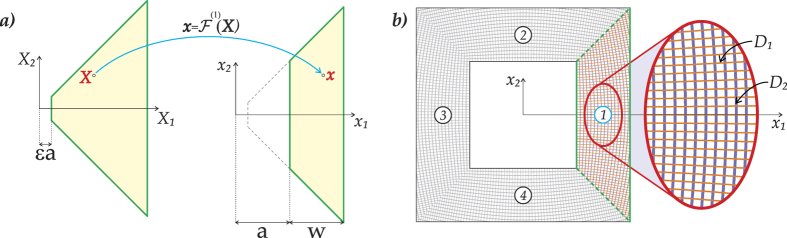
(**a**) The function 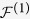 maps the undeformed trapezoidal region (1) to the deformed trapezoidal configuration. (**b**) A discrete lattice structure where the curved ligaments are oriented following the principal directions of the stiffness matrix for the continuum cloak.

Following Colquitt *et al*.^2^, we choose a regularised near-cloak, which is obtained by four push-out transformations applied to trapezoidal regions, as illustrated in Fig. 8a. The idea of regularisation is to map a domain with a small hole (e.g. a square of semi-width *εa* as in Fig. 8a) into another domain, whose exterior boundary is preserved while the interior boundary is expanded to the required finite size. For the regularised problem, we set the Neumann boundary condition on the boundary of the hole, which means (in the case of an elastic plate) the free edge boundary condition. As shown recently^11^, the correct choice of boundary condition is of vital importance in order to achieve cloaking, and in particular, replacement of the Neumann boundary condition with the Dirichlet condition, corresponding to a clamped boundary, would not be a feasible choice for cloaking of flexural waves in the Kirchhoff plate. This observation also answers a related question “Is the regularised cloak better than an imperfectly fabricated singular cloak”. For flexural waves in plates, the answer to this question is affirmative, for the reason that a regularised cloak allows for setting of the boundary condition on the interior contour of the cloak, and hence the asymptotic analysis follows (see^11^ for more details).

Here, as in^2^, the mapping is defined in such a way that 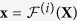 for each trapezoidal region (*j* = 1, 2, 3, 4) shown in Fig. 8a. In particular, the map for the trapezoidal region (1), with bases perpendicular to the *X*_1_–axis, is defined by the formula:


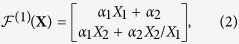


where


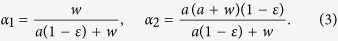


Here, 0 < *ε* ≪ 1 is the non-dimensional regularisation parameter parameter and uppercase letters denote the undeformed configuration, whilst lowercase letters denote the deformed configuration. Prior to the transformation, the interior boundary of the trapezoidal region (1) corresponds to *X*_1_ = *εa*. Following the transformation, the interior boundary of this trapezoidal region is moved to *x*_1_ = *a*. The outer boundary of the cloak is invariant with respect to the transformation, and for the region (1), it corresponds to *X*_1_ = *a* + *w*. The other three trapezoidal regions are transformed in the same way, subject to a rotation.

### Transformed equations after the cloaking transformation

The time-harmonic flexural deformations of a homogeneous thin elastic plate are governed by the Kirchhoff plate equation, which before the transformation has the form


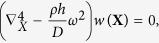


where *D* is the flexural rigidity, *w* is the transverse displacement amplitude, *ρ* is the mass density, *h* is the thickness of the plate, and *ω* is the radian frequency. After the application of the non-singular transformation (2) we have


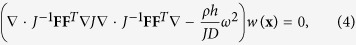


where 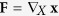 is the non-degenerate Jacobi matrix of the geometrical transformation, and *J* = det**F**. The above equation can be interpreted as the equation of an anisotropic pre-stressed plate, as discussed in^2^. In the context of asymptotic approximations, the paper^9^ discusses the configurations where the effects of pre-stress are small, and when an approximate factorisation of the transformed differential operator is possible.

Our goal is to construct an approximate cloak, which may be conveniently implemented experimentally. We observe that in the experiment for the continuous plate^17^, the authors assumed that the membrane waves are dominant and configured their radially symmetric approximate cloak accordingly. Although such an assumption may appear to be inappropriate, it has been demonstrated that, within a certain frequency range, the membrane waves are indeed dominant and the approximate cloaking effect is apparent. This approach is further reinforced for the square cloak by the observation made in^2^ that the principal directions of stiffness for the membrane cloak and the flexural cloak are exactly the same.

### The lattice approximation of the cloak

We consider an approximate cloak, realised using a discrete lattice structure with curved ligaments as derived in^2^. These elastic ligaments are aligned with the the principal directions of the stiffness matrix for the continuum cloak, as illustrated in Fig. 8b, and yield a structured flexural lattice system. The thin elastic ligaments may be treated as beams of rectangular cross section characterised by the bending stiffnesses *D*_1_ and *D*_2_ chosen in accordance with the analytical formulae from^2^


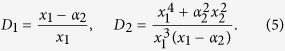


**Figure 9:**
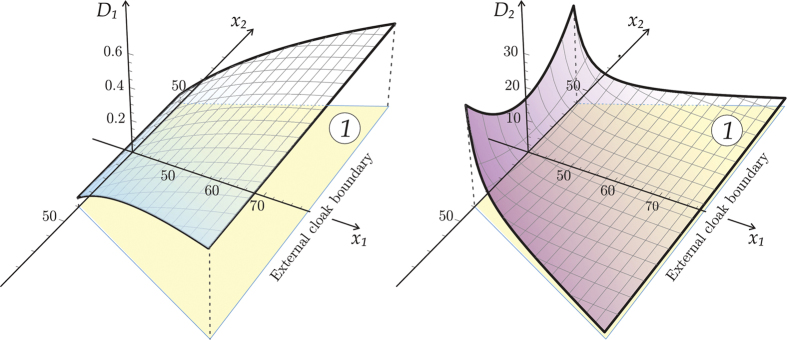
The required stiffnesses *D*_1_ and *D*_2_ for the cloak ligaments reported as function of *x*_1_ and *x*_2_.

These flexural rigidities are shown in Fig. 9 for the right-hand quadrant. Inside the cloak, *D*_1_ and *D*_2_ represent the stiffnesses in the principal directions of the locally orthotropic cloak constructed here. We note that on the interior boundary of the cloak the tangential rigidity *D*_2_ is much higher compared to the normal rigidity *D*_1_ and emphasise that these stiffnesses are finite on this boundary of the cloak (see Fig. 9).

Equations (5), together with the accompanying geometrical design of the cloak, provide the essential information for the experimental implementation discussed above.

## Concluding remarks

In this paper, we have presented a proof of concept design for a square invisibility cloak. Having constructed the cloak, we proceeded to examine its effectiveness using both a computational ABAQUS model as well as a real physical experiment. This novel design, proposed in an earlier theoretical paper^2^, was appealing due its simplicity and elegance, which made an experimental implementation feasible. The regularisation introduced into this design of cloak enables careful and precise implementation of boundary conditions on the interior boundary of the cloak in both the numerical simulations and the experiments.

The approximate cloak presented here proves to be efficient within a predicted frequency range, but the results become frequency sensitive as the frequency of the incident wave increases. This effect has been expected, and similar phenomena of high frequency sensitivity were noted in^17^.

The range of applications of the proposed cloaking device is wide and it covers, in particular, earthquake resistant systems, as well as novel designs of foundations of civil engineering structures.

## Additional Information

**How to cite this article**: Misseroni, D. *et al*. Cymatics for the cloaking of flexural vibrations in a structured plate. *Sci. Rep*. **6**, 23929; doi: 10.1038/srep23929 (2016).

## Supplementary Material

Supplementary Information

Supplementary Video S1
