# Supplementary material for: Cymatics for the cloaking of flexural vibrations in a structured plate
Source: Sci Rep. 2016 Apr 12;6:23929. doi: 10.1038/srep23929 (PMC4828639; doi:10.1038/srep23929)
Supplement: Supplementary Information [file srep23929-s1.pdf]

Title of the manuscript:

Cymatics for the cloaking of flexural vibrations in a structured plate

Author list:

Corresponding Author:
Dr. Diego Misseroni (diego.misseroni@unitn.it) (University of Trento)

Contributing Authors:
Dr. Daniel Colquitt , Prof. Alexander Movchan , Prof. Natalia Movchan , Prof. Ian Jones

Video legend:

Cymatics_movie.avi

Legend: Cymatics for the cloaking of flexural vibrations in a structured plate
